# Psychoeducational schema therapy for psoriasis and atopic dermatitis: a randomized controlled pilot crossover trial

**DOI:** 10.1111/ddg.70020

**Published:** 2026-03-19

**Authors:** Regina von Spreckelsen, Hila Emmert, Jan Hartmann, Matthias Hübenthal, Stephan Weidinger, Ulrich Mrowietz, Eva M. J. Peters, Sascha Gerdes

**Affiliations:** ^1^ Centre for Inflammatory Skin Diseases Clinic for Dermatology Venereology and Allergology, University Medical Centre Schleswig‐Holstein Campus Kiel Kiel Germany; ^2^ Psychoneuroimmunology Laboratory Clinic for Psychosomatics and Psychotherapy University Hospital Giessen Giessen Germany; ^3^ Department of Psychosomatic Medicine and Psychotherapy Charité Center for Internal Medicine and Dermatology Charité – Universitätsmedizin Berlin Free University of Berlin and Humboldt University of Berlin Berlin Germany

**Keywords:** atopic dermatitis, brief psychotherapy, depressive symptoms, integrated care, pilot trial, psoriasis, psychodermatology, psychoeducation, quality of life, schema therapy

## Abstract

**Background and objectives:**

Atopic dermatitis and psoriasis are chronic inflammatory skin diseases often linked to psychological stress. Integrative care models are lacking. This randomized pilot study aimed to develop and test a psychoeducational intervention for dermatology patients.

**Patients and methods:**

Patients with a PHQ‐2 score ≥ 3 at an outpatient inflammation center were randomized into an intervention or control group. The intervention group received three standardized educational sessions focusing on maladaptive schemas, coping strategies, psychoeducation, and emotion‐focused techniques (e.g. chair‐dialogues, imaginary rescripting).

**Results:**

19 patients received the intervention; 13 were in the control group. Post‐intervention, significant improvements were observed in dermatological quality of life (DLQI), subjective well‐being (WHO‐5), and depressive symptoms (PHQ‐9, BDI‐II). Psychological benefits were largely independent of disease severity (PASI, EASI). Qualitative feedback highlighted usability, learning specific techniques, and a trusting therapeutic relationship.

**Conclusions:**

A brief psychoeducational intervention significantly reduced psychological stress in dermatology outpatients. Further studies are needed to evaluate long‐term effects and broader implementation.

## INTRODUCTION

Chronic inflammatory skin diseases are highly comorbid with mental illnesses such as depression, anxiety disorders, or somatic stress disorders.^1^, ^2^, ^3^, ^4^ On average, around a third of all patients with skin diseases have psychological symptoms. Quality of life is even more frequently impaired and is also considered as a therapeutic outcome in pharmacological studies.^5^ However, a reduced health‐related quality of life (HRQoL) does not necessarily correlate with the severity of the disease, which may also contribute to the fact that patients' psychological stress often does not receive the appropriate attention in the somatic context.^6^ Patients often experience stigmatization instead of receiving support in coping with their disease. The resulting psychological stress plays an important role in the self‐management of dermatological patients.^7^ Poor mental health can also lead to a self‐reinforcing cycle where skin symptoms are exacerbated by stress and where reduced HRQoL can lead to reduced adherence to treatment.^8^

Stress and psychological strain are key factors for patients, and there is growing evidence that not only skin diseases favor mental illnesses, but that psychological stress also has an influence on the somatic hallmarks of the disease, such as itching.^9^, ^10^, ^11^, ^12^, ^13^

Psychological factors should be considered in the diagnosis and treatment of dermatological patients and be an integral component.^14^ This knowledge is reflected by the increasing research on psychotherapeutic interventions.^15^ New findings from psychoneuroimmunology also provide biopsychosocial concepts that emphasize the mutual connection between areas that were previously considered separately, skin diseases on the one hand and mental illnesses on the other.^16^ They support the modern approach of psychodermatology, which assumes that by using short questionnaires, patients suffering from stress can be identified at an early stage in their daily routine with little effort and promptly assigned suitable treatment options.^17^, ^18^ Since psychotherapeutic services are generally time‐consuming and the search for a suitable service can take a long time, innovative approaches are needed that can provide initial impetus in the outpatient sector with brief interventions.

Cognitive behavioral therapy (CBT) is a frequently investigated therapy method in this context.^19^, ^20^ Schema therapy (ST), a special form of CBT developed by Jeffrey Young,^21^ integrates cognitive, emotional and behavioral interventions as well as emotion‐ and behavior‐based approaches. A central element of schema therapy is the targeted development of a therapeutic relationship between therapist and patient. The symptoms, behavioral patterns and central biographical events are presented together on the flipchart in a way that is easily accessible to the patient with the help of the case conception.^22^ This approach addresses early maladaptive patterns arising from unmet basic emotional needs. Jeffrey Young's basic needs concept comprises four basic needs: Attachment, autonomy and self‐esteem as well as play and spontaneity.^21^ An individual case conception is developed together with the patient to understand and change dysfunctional coping behavior (e.g. avoidance behavior due to skin diseases).^23^, ^24^ In a previous study, we demonstrated that a psychotherapeutic intervention based on schema‐therapeutic constructs and the identification of experienced stigmatization was associated with an improvement in patients’ quality of life.^25^ As psychotherapeutic interventions have not been regularly offered or are difficult to access for mentally stressed patients without manifest depression in everyday outpatient dermatological care, we have developed and implemented a brief, manualized, psychoeducational intervention with schema therapy elements with three manual and protocol‐based sessions – the *Ultra‐Intensive Short‐Term Schema Therapy* (ULTRA‐ISTST).

The present study was designed as a pilot trial to assess the feasibility, acceptability, and preliminary clinical effects of the Ultra‐ISTST in an outpatient dermatology context. As a pilot study, it aimed to generate essential data on recruitment, screening, adherence, and effect size estimates to inform the design and conduct of a future definitive randomized controlled trial. The choice of a crossover design allowed all participants, including those initially randomized to the control group, to benefit from the intervention, while also enabling within‐study comparisons between groups. Specific objectives were to evaluate changes in depressive symptoms, quality of life, and well‐being following Ultra‐ISTST, to explore whether these effects were independent of dermatological disease severity, and to assess patient‐perceived value of the intervention.

## MATERIAL AND METHODS

### Study design

This investigation was conducted as a single‐center, randomized, controlled pilot trial with a crossover design. The trial had an exploratory framework, aiming to evaluate the feasibility and preliminary effects of a brief psychological‐educational intervention (Ultra‐ISTST) in adult patients with psoriasis vulgaris (PsV) or atopic dermatitis (AD) who exhibited elevated depressive symptoms. Participants were allocated to study arms in a 1:1 ratio. Patients or members of the public were not involved in the design, conduct, reporting, or dissemination plans of this research.

Recruitment and study conduct took place between 14 June 2021 and 14 August 2021 at the Center for Inflammatory Skin Diseases, University Hospital Schleswig‐Holstein (UKSH), Campus Kiel, Germany. The trial ended as planned after completion of recruitment and follow‐up; no early termination occurred. Eligible participants were adult patients with a confirmed diagnosis of PsV or AD, established by a dermatologist at least three months prior to study enrolment. Screening for potential eligibility was performed consecutively during routine outpatient registration using the Patient Health Questionnaire‐2 (PHQ‐2). ^26^

Inclusion criteria were age ≥ 18 years, a Patient Health Questionnaire‐2 (PHQ‐2) score ≥ 3, and provision of written informed consent. Exclusion criteria included manifest mental illness (for example, major depressive disorder or psychotic disorders), insufficient proficiency in the German language, pregnancy or breastfeeding, acute suicidal ideation, and current drug or alcohol abuse. Potential participants meeting the eligibility criteria were provided with detailed study information and gave written informed consent before randomization.

### Interventions

Participants randomized to the intervention group received, in addition to guideline‐based dermatological care, three individual sessions of a manualized, protocol‐based intervention (Ultra‐ISTST), delivered by trained psychotherapists at 2‐week intervals. Each session lasted approximately 50 minutes and incorporated elements from schema therapy, cognitive behavioral therapy, and emotion‐focused approaches, including techniques such as chair dialogues and imagery rescripting.^15^ A central component of the intervention was the joint development of an individualized case conceptualization with the participant (Table 1).

**TABLE 1:** Overview of the manualized three‐session intervention.

Session	Topic	Contents
*1*	***Relationship building***	
	Presentation Ultra ISTST Intervention	Welcome, introduction
	Introduction of individual case conception	Specific description of the stressful life situation in the context of the dermatological disease
		Focus work on the flipchart: adult mode; knowledge and recognition of own resources and functional behavior in everyday life
		Exercise a: Imagination: Encountering the adult and child self
		Exercise to do at home:
		Recognizing and documenting positive everyday situations, reviewing the case conception in everyday life
*2*	***Individual case conception in the context of the dermatological disease***	
	Focus on basic needs	Focus work on the flipchart: Child mode: Knowledge and recognition of general core child needs in comparison to your own happy but also vulnerable child parts
	Focus on dysfunctional coping strategies	Focus work on the flipchart: dysfunctional coping strategies: Knowledge and recognition of your own coping patterns
		Exercise b: Create pro/contra lists regarding own coping strategies
		Exercise to do at home a: Pleasure exercise
		Exercise to do at home b: Self‐observation and recognizing individual coping strategies in the everyday context
*3*	***Individual case conception***	
	Focus on "your own inner critic (parent mode)	Focus work on the flipchart: “your own inner critic” (parent mode); recognizing self‐deprecation and self‐punishment and placing them in a biographical and current context
		Exercise c: Instructions for self‐conducting a chair dialogue against self‐devaluation and self‐punishment
	Conclusion, farewell	Summary of important key points of the individual case concept and transfer to everyday life

Participants in the control group received treatment‐as‐usual (TAU), consisting solely of guideline‐based dermatological care, without any psychotherapeutic intervention. In both groups, dermatological treatment decisions were made independently of study participation, in accordance with current clinical guidelines, and could range from topical therapy to systemic biologic therapy. The psychometric questionnaires used in the study (e.g., DLQI, WHO‐5, PHQ‐9, BDI‐II) were not part of routine care and were therefore considered part of the study intervention package.

Following completion of the control phase, participants in the control group were offered the opportunity to take part in the Ultra‐ISTST intervention under identical conditions, as per the crossover design.

### Outcomes

The primary outcome was the change in PHQ‐2 score from baseline (V1) to post‐intervention (V4).

Secondary outcomes included the Dermatology Life Quality Index (DLQI),^27^ the WHO‐5 Well‐Being Index,^28^ the Beck Depression Inventory II (BDI‐II),^29^ and the Patient Health Questionnaire‐9 (PHQ‐9). Disease severity was assessed using the Psoriasis Area and Severity Index (PASI)^30^ for PsV patients, and the Eczema Area and Severity Index (EASI) and SCORing Atopic Dermatitis (SCORAD) for AD patients.^31^, ^32^

Outcome assessments were conducted at baseline (V1) and after completion of the intervention (V4). For the intervention group, an additional follow‐up was performed three months after completion of the intervention (V5). All measurement instruments were administered in their validated German versions, and scoring procedures, aggregation methods, ranges, and clinical cut‐offs followed the respective original publications.

### Randomization and allocation concealment

Randomization was performed using a simple randomization procedure without stratification or blocking. For each available study slot, an opaque, sealed, identical envelope containing the group assignment (“intervention” or “control”) was prepared. All envelopes were placed in an opaque container and thoroughly mixed. Immediately after a participant provided informed consent, one envelope was drawn by an independent member of the study staff who was not involved in recruitment, intervention delivery, or statistical analysis. This procedure ensured full allocation concealment until the moment of assignment. As this was a pilot study, the sample size was determined pragmatically, based on available personnel, time, and therapy capacity. No formal sample size calculation was performed, as the primary aim was to assess feasibility and to generate preliminary effect size estimates to inform the design of a future definitive trial. No interim analyses or stopping guidelines were planned or implemented.

### Blinding

Due to the nature of the intervention, neither participants nor treating clinicians could be blinded to group allocation. However, statistical analyses were performed by independent statisticians who were blinded to individual patient identities and worked exclusively with anonymized group codes.

### Statistical analysis

Descriptive statistics were calculated for all variables. Between‐group comparisons of continuous outcomes were conducted using the Wilcoxon rank‐sum test. Values outside 1.5 times the interquartile range were considered outliers and excluded from analysis. Additionally, linear mixed‐effects models with random intercepts for patients were fitted (model formula: score ∼ visit*group + (1|patient)) and compared with null models (score ∼ (1|patient)) using analysis of variance (ANOVA). Effect sizes and 95% confidence intervals were calculated. Analyses were performed using R version 4.3.1, with mixed models fitted using the lme4 package.^33^, ^34^ All statistical tests were two‐sided, and p values below 0.05 were considered statistically significant.

For the main between‐group analyses, all participants who received the Ultra‐ISTST intervention during the study period were analyzed together as the intervention group (n = 19). This included eleven participants initially randomized to the intervention arm and eight participants from the control group who received the intervention during the crossover phase. These were compared with participants in the control group (n = 13) who did not receive the intervention during the study period. Analyses were conducted on complete cases only. No subgroup or sensitivity analyses were prespecified or conducted.

This approach was chosen deliberately to maximize the number of participants in the intervention group, thereby increasing the precision of effect size estimates for this pilot trial. We acknowledge that this analysis departs from a pure randomized comparison and may introduce bias; therefore, the results should be interpreted with caution and not as definitive evidence of efficacy.

The full study protocol and the manualized intervention materials are available from the corresponding author upon reasonable request (Table 1).

### Assessment of harms

No formal adverse event monitoring was performed, as the intervention was non‐pharmacological and classified as low risk. At each study visit, the treating dermatologist assessed the participant's general health status. No intervention‐related adverse events were reported.

### Ethical approval

The study was approved by the Ethics Committee of the Medical Faculty of Kiel University (reference number D 473/21). The trial was not registered in a public trial registry, as it did not involve a medicinal product or medical device and was not subject to mandatory registration under applicable local regulations.

## RESULTS

The screening revealed that a total of 141 patients out of 885 had a PHQ‐2 score of ≥ 3 points. Of these positively screened patients, 26 (18.5%) had known depression and were not eligible for our study based on the inclusion criteria. Of the remaining positively screened patients, 79 (56.0%) were open to intervention, while 36 (25.5%) were not interested. Of the 24 available Ultra‐ISTST treatment places, eleven patients were randomized to the intervention group and 13 to the control group (Figure 1). The remaining positively screened patients were informed of the results of their screening questionnaire and, where appropriate, advised to seek further assessment from their general practitioner or a mental health professional. After completion of the intervention, an additional eight people from the control group took up the intervention offer, so that a total of 19 patients received the intervention. All between‐group comparisons presented below are based on these two groups (Intervention n = 19; Control n = 13). The sample characteristics of all included patients are shown in Table 2. Within the study period no patient had changes to their dermatological therapy, including systemic and topical treatments.

**FIGURE 1:**
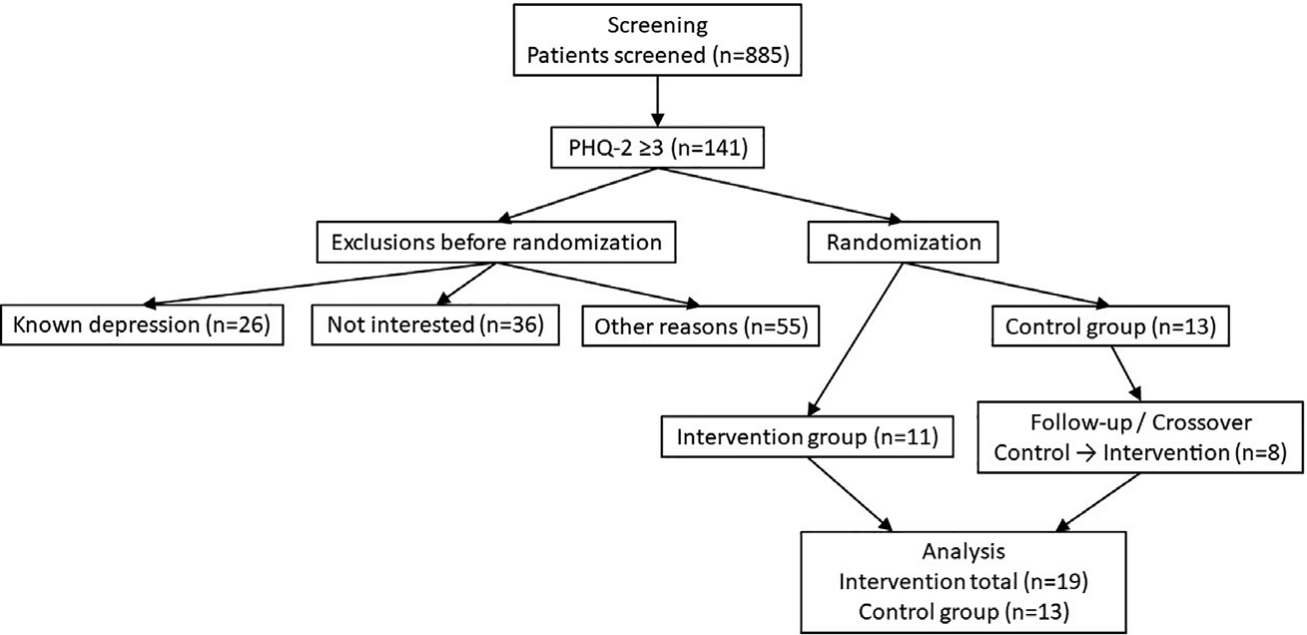
CONSORT flow diagram illustrating participant screening, exclusions, randomization, follow‐up, and analysis.

**TABLE 2:** Sample characteristics.

Patients	Total n = 24
Age in years (mean ± SD)	47.8 ± 13.3
Gender; female n	13
topical therapy; n	4
Systemic oral therapy; n	5
Systemic s.c. therapy; n	15
PHQ‐9 at baseline (mean ± SD)	10.3 ± 4.47
*Psoriasis; n*	***18***
Intervention group	
PASI at baseline (mean ± SD)	2.8 ± 1.5
PASI at end of study (mean ± SD)	2.7 ± 1.9
Control group	
PASI at baseline (mean ± SD)	5.7 ± 4.3
PASI at end of study (mean± SD)	3.5 ± 3.4
*Atopic dermatitis; n*	***6***
Intervention group	
EASI at baseline (mean± SD)	7.8 ± 9.1
EASI at end of study (mean± SD)	8.4 ± 10.0
Control group	
EASI at baseline (mean± SD)	8.0 ± 7.2
EASI at end of study (mean± SD)	3.4 ± 0.4

*Abbr*.: EASI, Eczema Area and Severity Index; PASI, Psoriasis Area and Severity Index; PHQ‐9, Patient Health Questionnaire‐9; s.c., subcutaneous; SD, standard deviation

The improvement of the PHQ‐2 was the main target criterion of the present study. In addition, the effect of the Ultra‐ISTST intervention was analyzed using the psychometric questionnaires DLQI, PHQ‐9, WHO‐5, BDI‐II. The intervention group showed a statistically significant improvement in the PHQ‐2 (p = 0.002) and DLQI (p = 0.014) after the intervention (V4) compared to the baseline visit (V1) (Figure 2), whereas the only significantly affected score within the control group was PHQ‐2 (p = 0.034). To determine whether the improvement in quality of life continued after the intervention, the patients were surveyed again 3 months after the end of the intervention (V5). The analysis of all the psychological questionnaire values analyzed demonstrated that the significant improvement at the end of the intervention in the PHQ‐2 (p = 0.001) continued 3 months after the intervention and in the case of the WHO‐5 (p = 0.015) had only improved significantly in relation to the baseline after 3 months (Figure 2).

**FIGURE 2:**
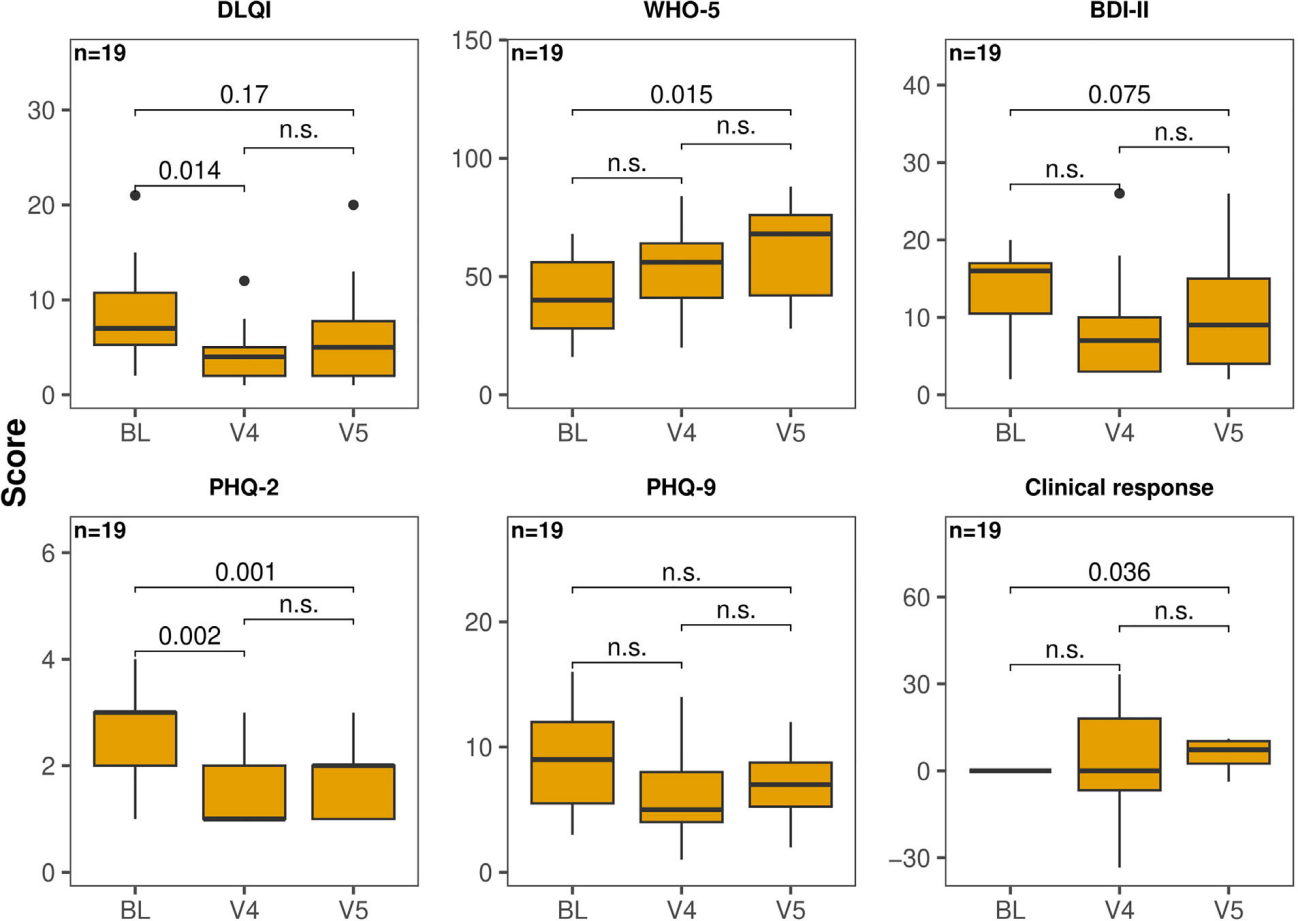
Results of questionnaire assessments in the intervention group (n = 19) over the course of the intervention. DLQI, WHO‐5, BDI‐II, PHQ‐2, and PHQ‐9 were assessed using validated instruments. Dermatological responses were evaluated at V4 (6 weeks after baseline) and V5 (12 weeks after baseline) and are presented as percentage change from baseline. P values represent results of the Wilcoxon rank‐sum test between time points. n = number of participants.

The direct comparison of the intervention group with the control group after the intervention (V4) showed significantly better values in the PHQ‐2 (p = 0.005), DLQI (p = 0.003), BDI‐II (p = 0.001) and PHQ‐9 (p = 0.020) in the intervention group compared to the control group (Figure 3). To determine whether the improved quality of life was associated with an improvement in the clinical severity of the disease, the improvement in clinical symptoms was analyzed using the percentage change in the PASI (in psoriasis patients) or the EASI (in AD patients). It was found that clinical symptoms improved in both the intervention and the control group compared to baseline, but there was a significantly greater improvement in the intervention group compared to the control group at visit 4 (p = 0.039) (Figure 3).

**FIGURE 3:**
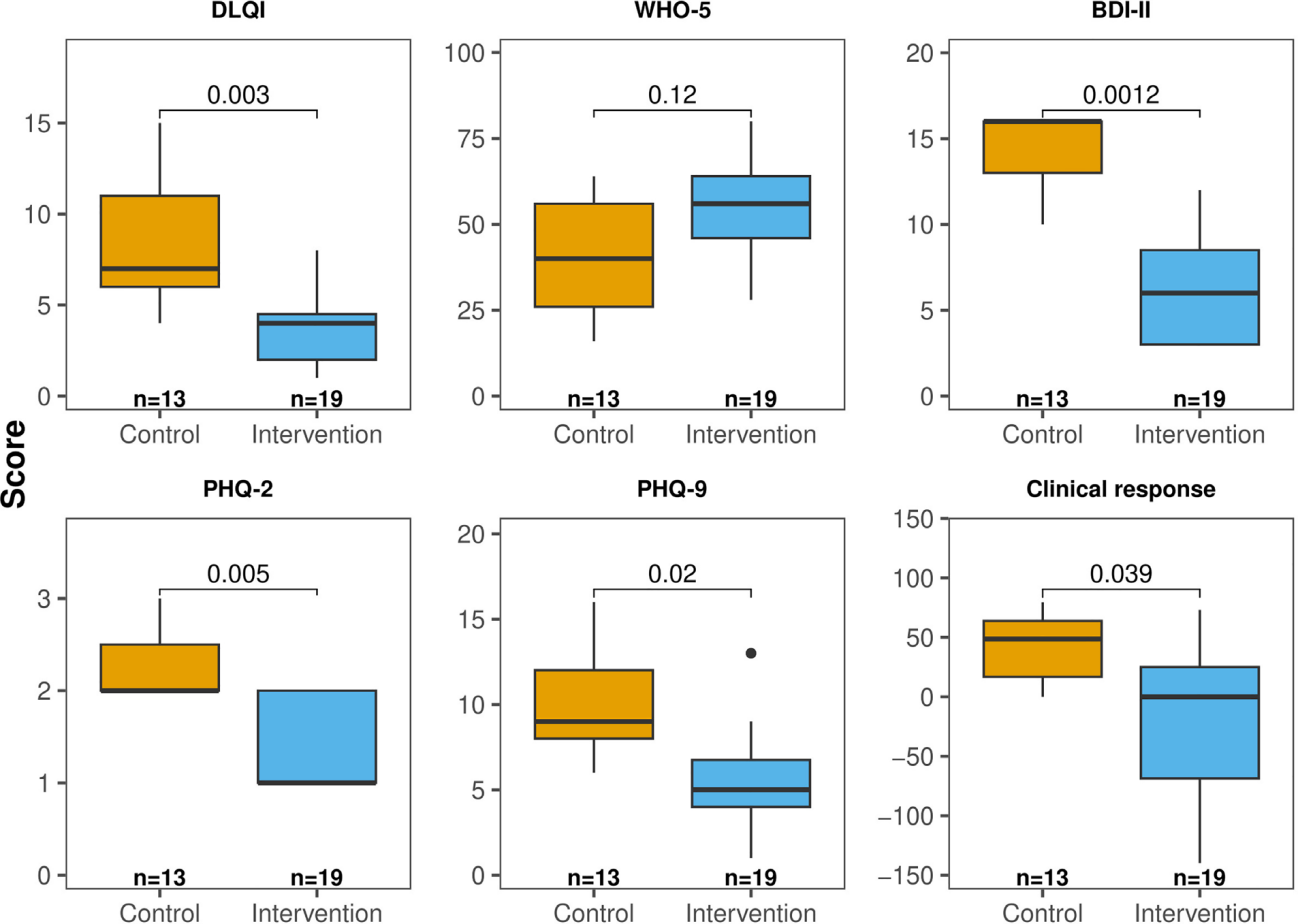
Comparison of psychological questionnaire outcomes and clinical symptom severity at V4 between the control group (n = 13) and the intervention group (n = 19). DLQI, WHO‐5, BDI‐II, PHQ‐2, and PHQ‐9 were assessed using validated instruments. Dermatological response was evaluated at V4 as percentage change from baseline. Clinical severity was assessed using the Eczema Area and Severity Index (EASI) for atopic dermatitis and the Psoriasis Area and Severity Index (PASI) for psoriasis. P values represent results of the Wilcoxon rank‐sum test between groups. n = number of participants.

Linear mixed models (LMM) were used to determine the dependent associations of the outcome variables of the questionnaires with the implementation of the intervention. The statistical analysis of the LMM showed that the psychotherapeutic intervention had a significant effect on the PHQ‐2 as well as on the DLQI, the WHO‐5, the BDI‐II and the PHQ‐9 (Figure 4). In the next step, these models were expanded to include clinical response (percentage change in EASI/PASI) as dependent variable. It was shown that the observed change in somatic findings during the intervention itself had no significant influence on the improvement in mental health and quality of life (Figure 5). Thus, it could be shown that the improvement in depressive symptoms, quality of life and psychological well‐being through the Ultra‐ISTST was independent of an improvement in the clinical picture.

**FIGURE 4:**
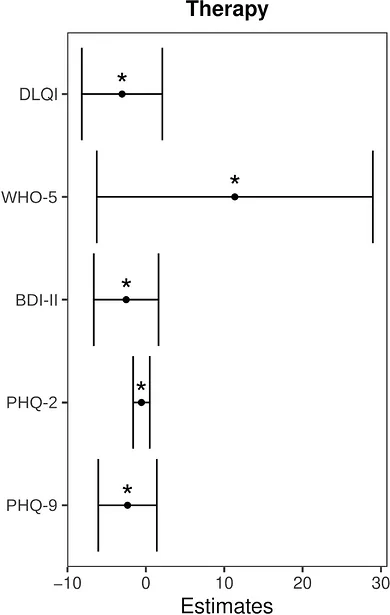
Effect sizes of the intervention on questionnaire outcomes at V4. Effect sizes were estimated using linear mixed models. Statistical significance reflects comparison of the intervention model (score ∼ group × visit + [1 | patient]) with the null model (score ∼ [1 | patient]) using analysis of variance. *p < 0.05; n.s., not significant.

**FIGURE 5:**
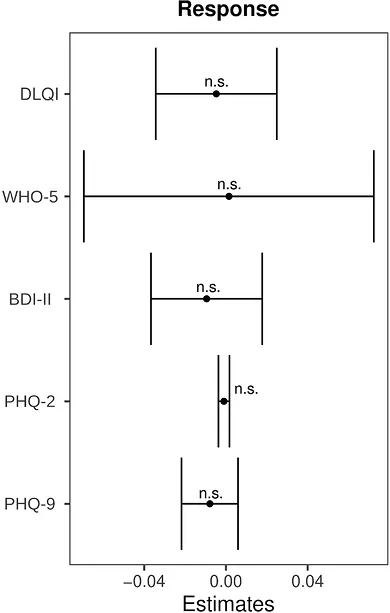
Effect sizes of clinical response on questionnaire outcomes at V4. Effect sizes were estimated using linear mixed models. Statistical significance reflects comparison of the response model (score ∼ group affiliation × visit + clinical response + [1 | patient]) with the treatment model (score ∼ group affiliation × visit) using analysis of variance. *p < 0.05; n.s., not significant.

To additionally assess the effectiveness of the Ultra‐ISTST, the quality of the intervention was reviewed and descriptively evaluated after the end of the last individual session using a specially established and standardized evaluation form (Figure 6). A total of 46 statements were identified from the free‐text responses and assigned to four main categories with corresponding subcategories. Of these, 24 statements pertained to the main category “suitability for everyday use” of the intervention. The participants focused primarily on raising awareness of individual problems, concretization and finding solutions together. One participant stated: “I used the individualized case formulation to identify my personal stressors, enabling me to manage them more effectively in the future […] and to be more mindful of them in my daily life.” A total of nine statements were identified within the main category “therapist–patient relationship,” of which six were assigned to the subcategory “feeling safe and secure.” Eight statements were identified for the main category “learning specific techniques.” Participants highlighted core schema‐therapeutic techniques, such as chair work and imagery. In addition, five statements reflected strengthened self‐esteem, for example: “[…] I was reminded of my strengths” (Table 3).

**FIGURE 6:**
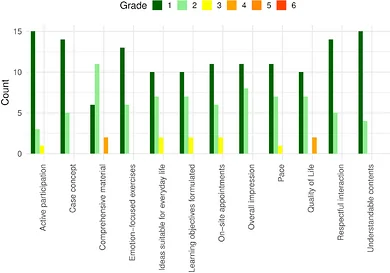
Patient‐rated evaluation of the intervention using the German school grading system (1 = very good, 2 = good, 3 = satisfactory, 4 = sufficient, 5 = poor, 6 = unsatisfactory).

**TABLE 3:** Distribution of statements within the category system.

Main category	Subcategory	Statements (n)	Statements (%)
Suitability of the sessions for everyday use	Implementing the content in everyday life	4	16,7
	Awareness of individual problems	9	37,5
	Concretization and solution finding	7	29,2
	Generally sensible selection of topics	4	16,7
	*Total*	24	100
Strengthening self‐esteem	Recognized own strengths through reflection	5	100
Learning specific techniques	Breathing exercises	1	12,5
	Stress reduction training	1	12,5
	Chair dialogue	3	37,5
	Imaginations	2	25,0
	Relaxation exercises	1	12,5
	*Total*	8	100
Therapist‐patient relationship	Felt accepted and valued	1	11,1
	Felt safe and secure	6	66,7
	Opportunity to open up and speak out	2	22,2
	*Total*	9	100

No harm or unintended effects were observed in either group during the study period.

## DISCUSSION

Various psychotherapeutic intervention formats for patients with chronic skin diseases, ranging from training programs, psychodynamic therapies, cognitive behavioral therapies, mindfulness‐based interventions, stress management and relaxation techniques to systemic approaches and long‐term psychotherapy in guideline procedures, have been described.^19^, ^35^, ^36^, ^37^ Here we describe ULTRA‐ISTST, a behavioral therapy approach with the aim of offering a low‐threshold service for mentally stressed patients without manifest depression in a dermatological context. The short‐term intervention, consisting of three manual‐based 50‐minute therapy sessions, showed significant efficacy in improving depressive symptoms and reduced quality of life in stressed patients compared to a control group.

For comprehensive care of patients with dermatological diseases, economical screening for psychological symptoms in an outpatient somatic care context is desirable. Gerdes et al. were able to show that the Whooley questions are an easy‐to‐use method for screening for depression in psoriasis patients.^18^ In addition, Köster et al. were able to implement the freely available Patient Health Questionnaire‐4 (PHQ‐4) in the electronic patient file for the early detection of mental disorders in the inpatient context, thus enabling a psychosomatic consultation for those affected in a timely manner.^38^ Based on these results, we used the freely available PHQ‐2 in our pilot study to identify stressed patients. We were again able to show that it is possible to detect psychologically distressed patients in an outpatient context. With the help of the Ultra‐ISTST, we were able to offer these patients low‐threshold treatment immediately after screening as part of the dermatological consultation, from which a considerable proportion were able to benefit significantly.

Psychosocial factors and illnesses pose a particular challenge. Referral of dermatology outpatients with stress‐related symptoms to further care is often challenging for treating dermatologists, partly due to the lack of established interdisciplinary care structures.^39^ The integration of somatic and psychotherapeutic treatment is of current importance and is reflected in the implementation of psychodermatology. In chronic inflammatory diseases, a neuroimmunological connection between somatic inflammation and psychological comorbidity has also been demonstrated.^40^ Therefore, a multidisciplinary approach to the treatment of chronic inflammatory skin patients is favored today.^41^

Short‐term interventions, particularly for patients with mild stress without manifest mental illness, can close a gap in care between outpatient needs and standard care as part of guideline psychotherapy. The Ultra‐ISTST pilot study showed a significant improvement in quality of life and a reduction in depressive symptoms after three treatment appointments, irrespective of an improvement in skin findings as part of standard therapy. However, the concept presented here should not be considered a substitute for established therapeutic approaches but rather a low‐threshold support program that can be integrated into primary dermatological care. Basic psychosomatic care can play a decisive role here.^42^ Basic psychosomatic care is not a mandatory component of dermatology specialist training but may be obtained on an optional basis when required for billing of basic psychosomatic services.^43^ The manual‐based and time‐limited intervention conducted in our study could serve as a basis for interested dermatologists who wish to offer a quality‐assured short‐term intervention in their everyday practice. A particular strength of our Ultra‐ISTST is the measured sustainability of the intervention. Patients in the intervention group showed a constant improvement in their depressive symptoms and quality of life even 3 months after the end of the intervention. The patients stated that they benefited above all from becoming aware of their own stress factors and learning specific techniques in a safe and secure environment. This holistic approach provided patients with a better understanding of the stigmatization they experienced.^44^ Based on the assumption that patients with skin diseases develop dysfunctional coping strategies under stress, understanding their own case conceptualization can motivate patients to better integrate concrete alternative ideas for dealing with new challenges into their lives.^45^

One important finding of this study was that the observed improvements in mental health and quality of life were largely independent of changes in dermatological disease severity. No harm or unintended effects were observed in either group during the study period. This suggests that Ultra‐ISTST may address specific psychosocial needs that are not directly related to clinical skin status.

This pilot study has several limitations. First, the primary between‐group analyses combined all participants who received the intervention, irrespective of their original randomization, in order to maximize the sample size for effect size estimation. While appropriate in the context of a pilot trial, this approach departs from a strict randomized comparison and may overestimate treatment effects due to potential selection bias. Second, it was conducted in a specialized outpatient clinic, which may limit the generalizability of the findings to other care settings. Third, the trial was unblinded and did not include an active control intervention, meaning that non‐specific treatment effects cannot be ruled out. Fourth, the sample size was small and pragmatically chosen without a formal power calculation, limiting statistical power to detect smaller effects. Fifth, although no intervention‐related adverse events were reported, adverse events were not systematically monitored, although the general health status of participants was checked at each visit.

The patient population was drawn from a tertiary dermatology center in Germany, and most participants were already receiving guideline‐based dermatological care. While the manualized nature of the intervention may allow adaptation to broader contexts, its feasibility in primary care or in settings without access to psychotherapeutic expertise remains to be evaluated.

### Implications for future research

As a pilot trial, the main objective was to assess feasibility and inform the design of a larger, definitive randomized controlled trial. Future studies should consider a multi‐center design, include an active control arm, implement longer‐term follow‐up for both groups, and systematically monitor potential adverse events.

## CONFLICT OF INTEREST STATEMENT

None.
